# Microbial Biogeography of Public Restroom Surfaces

**DOI:** 10.1371/journal.pone.0028132

**Published:** 2011-11-23

**Authors:** Gilberto E. Flores, Scott T. Bates, Dan Knights, Christian L. Lauber, Jesse Stombaugh, Rob Knight, Noah Fierer

**Affiliations:** 1 Cooperative Institute for Research in Environmental Science, University of Colorado, Boulder, Colorado, United States of America; 2 Department of Computer Science, University of Colorado, Boulder, Colorado, United States of America; 3 Department of Chemistry and Biochemistry, University of Colorado, Boulder, Colorado, United States of America; 4 Howard Hughes Medical Institute, University of Colorado, Boulder, Colorado, United States of America; 5 Department of Ecology and Evolutionary Biology, University of Colorado, Boulder, Colorado, United States of America; Auburn University, United States of America

## Abstract

We spend the majority of our lives indoors where we are constantly exposed to bacteria residing on surfaces. However, the diversity of these surface-associated communities is largely unknown. We explored the biogeographical patterns exhibited by bacteria across ten surfaces within each of twelve public restrooms. Using high-throughput barcoded pyrosequencing of the 16 S rRNA gene, we identified 19 bacterial phyla across all surfaces. Most sequences belonged to four phyla: *Actinobacteria*, *Bacteriodetes*, *Firmicutes* and *Proteobacteria*. The communities clustered into three general categories: those found on surfaces associated with toilets, those on the restroom floor, and those found on surfaces routinely touched with hands. On toilet surfaces, gut-associated taxa were more prevalent, suggesting fecal contamination of these surfaces. Floor surfaces were the most diverse of all communities and contained several taxa commonly found in soils. Skin-associated bacteria, especially the *Propionibacteriaceae*, dominated surfaces routinely touched with our hands. Certain taxa were more common in female than in male restrooms as vagina-associated *Lactobacillaceae* were widely distributed in female restrooms, likely from urine contamination. Use of the SourceTracker algorithm confirmed many of our taxonomic observations as human skin was the primary source of bacteria on restroom surfaces. Overall, these results demonstrate that restroom surfaces host relatively diverse microbial communities dominated by human-associated bacteria with clear linkages between communities on or in different body sites and those communities found on restroom surfaces. More generally, this work is relevant to the public health field as we show that human-associated microbes are commonly found on restroom surfaces suggesting that bacterial pathogens could readily be transmitted between individuals by the touching of surfaces. Furthermore, we demonstrate that we can use high-throughput analyses of bacterial communities to determine sources of bacteria on indoor surfaces, an approach which could be used to track pathogen transmission and test the efficacy of hygiene practices.

## Introduction

More than ever, individuals across the globe spend a large portion of their lives indoors, yet relatively little is known about the microbial diversity of indoor environments. Of the studies that have examined microorganisms associated with indoor environments, most have relied upon cultivation-based techniques to detect organisms residing on a variety of household surfaces [1]–[5]. Not surprisingly, these studies have identified surfaces in kitchens and restrooms as being hot spots of bacterial contamination. Because several pathogenic bacteria are known to survive on surfaces for extended periods of time [6]–[8], these studies are of obvious importance in preventing the spread of human disease. However, it is now widely recognized that the majority of microorganisms cannot be readily cultivated [9] and thus, the overall diversity of microorganisms associated with indoor environments remains largely unknown. Recent use of cultivation-independent techniques based on cloning and sequencing of the 16 S rRNA gene have helped to better describe these communities and revealed a greater diversity of bacteria on indoor surfaces than captured using cultivation-based techniques [10]–[13]. Most of the organisms identified in these studies are related to human commensals suggesting that the organisms are not actively growing on the surfaces but rather were deposited directly (i.e. touching) or indirectly (e.g. shedding of skin cells) by humans. Despite these efforts, we still have an incomplete understanding of bacterial communities associated with indoor environments because limitations of traditional 16 S rRNA gene cloning and sequencing techniques have made replicate sampling and in-depth characterizations of the communities prohibitive. With the advent of high-throughput sequencing techniques, we can now investigate indoor microbial communities at an unprecedented depth and begin to understand the relationship between humans, microbes and the built environment.

In order to begin to comprehensively describe the microbial diversity of indoor environments, we characterized the bacterial communities found on ten surfaces in twelve public restrooms (six male and six female) in Colorado, USA using barcoded pyrosequencing of the 16 S rRNA gene. Compared to other indoor environments, public restrooms offer a unique setting to explore microbial diversity because of the activities that take place there and the high frequency of use by individuals with different hygienic routines. These features are likely to have strong influences on the types of bacteria observed on restroom surfaces. Our objectives for this study were to (i) determine the composition of microbial communities associated with common restroom surfaces, (ii) determine if different surfaces host different communities, and (iii) determine sources of bacteria in restroom environments and how the relative importance of these sources varies across specific locations within restrooms.

## Materials and Methods

### Sampling, DNA extraction and pyrosequencing

Ten surfaces (door handles into and out of the restroom, handles into and out of a restroom stall, faucet handles, soap dispenser, toilet seat, toilet flush handle, floor around the toilet and floor around the sink) in six male and six female restrooms evenly distributed across two buildings on the University of Colorado at Boulder campus were sampled on a single day in November 2010. Surfaces where sampled using sterile, cotton-tipped swabs as described previously [14], [15]. As the 12 restrooms were nearly identical in design, we were able to swab the same area at each location between restrooms. In order to characterize tap water communities as a potential source of bacteria, 1 L of faucet water from six of the restrooms (each building having the same water source for each restroom sampled) was collected and filtered through 0.2 µm bottle top filters (Nalgene, Rochester, NY, USA). Genomic DNA was extracted from the swabs and filters using the MO BIO PowerSoil DNA isolation kit following the manufacturer's protocol with the modifications of Fierer *et al*. [14]. A portion of the 16 S rRNA gene spanning the V1–V2 regions was amplified using the primer set (27 F/338R), PCR mixture conditions and thermal cycling conditions described in Fierer *et al*. [15]. PCR amplicons of triplicate reactions for each sample were pooled at approximately equal amounts and pyrosequenced at 454 Life Sciences (Branford, CT, USA) on their GS Junior system. A total of 337,333 high-quality partial 16 S rRNA gene sequences were obtained from 101 of the 120 surface samples collected, averaging approximately 3,340 sequences per sample (ranging from 513–6,771) (Table S1) in 4 GS Junior runs, with the best run containing 116,004 high-quality reads. An additional 16,416 sequences (ranging from 2161–5084 per sample) were generated for five of the six water samples collected for source tracking analysis. Each sample was amplified with a unique barcode to enable multiplexing in the GS Junior runs. The barcoded sequencing reads can be separated by data analysis software providing high confidence in assigning sequencing read to each sample. Sequence data generated as part of this study is available upon request by contacting the corresponding author.

### Sequence analysis

All sequences generated for this study and previously published data sets used for source tracking (see below) were processed and sorted using the default parameters in QIIME [16]. Briefly, high-quality sequences (>200 bp in length, quality score >25, exact match to barcode and primer, and containing no ambiguous characters) were trimmed to 300 bp and clustered into operational taxonomic units (OTUs) at 97% sequence identity using UCLUST [17]. Representative sequences for each OTU were then aligned using PyNAST [18] against the Greengenes core set [19] and assigned taxonomy with the RDP-classifier [20]. Aligned sequences were used to generate a phylogenetic tree with FastTree [21] for both alpha- (phylogenetic diversity, PD) [22] and beta-diversity (unweighted UniFrac) [23] metrics. The unweighted UniFrac metric, which only accounts for the presence/absence of taxa and not abundance, was used to determine the phylogenetic similarity of the bacterial communities associated with the various restroom surfaces. The UniFrac distance matrix was imported into PRIMER v6 where principal coordinate analysis (PCoA) and analysis of similarity (ANOSIM) were conducted to statistically test the relationship between the various communities [24]. In order to eliminate potential biases introduced by sampling depth, all samples (including those used in source tracking) were rarified to 500 sequences per sample for taxonomic, alpha-diversity (PD), beta-diversity (UniFrac) and source tracking comparisons.

### Source tracking

To determine the potential sources of bacteria on restroom surfaces and how the importance of different sources varied across the sampled locations, we used the newly developed SourceTracker software package [25]. The SourceTracker model assumes that each surface community is merely a mixture of communities deposited from other known or unknown source environments and, using a Bayesian approach, the model provides an estimate of the proportion of the surface community originating from each of the different sources. When a community contains a mixture of taxa that do not match any of the source environments, that portion of the community is assigned to an “unknown” source. Potential sources we examined included human skin (n = 194), mouth (n = 46), gut (feces) (n = 45) [26] and urine (n = 50), as well as soil (n = 88) [27] and faucet water (n = 5, see above). For skin communities, sequences collected from eight body habitats (palm, index finger, forearm, forehead, nose, hair, labia minora, glans penis) from seven to nine healthy adults on four occasions were used to determine the average community composition of human skin [26]. The mouth (tongue and cheek swabs), gut and urine communities were determined from the same individuals although the urine-associated communities were not published in the initial report of these data [26]. While urine is generally considered to be sterile, it does pick up bacteria associated with the urethra and genitals [28], [29]. The average soil community was determined from a broad diversity of soil types collected across North and South America [27].

## Results and Discussion

**Figure 1:**
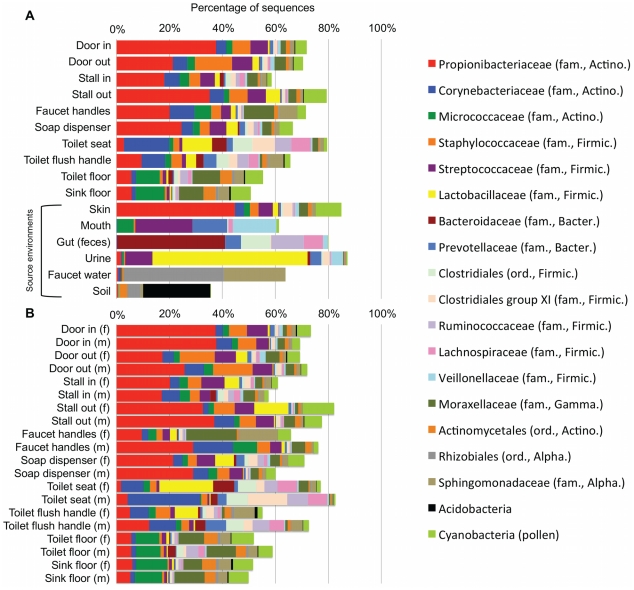
Taxonomic composition of bacterial communities associated with public restroom surfaces. (**A**) Average composition of bacterial communities associated with restroom surfaces and potential source environments. (**B**) Taxonomic differences were observed between some surfaces in male and female restrooms. Only the 19 most abundant taxa are shown. For a more detailed taxonomic breakdown by gender including some of the variation see Supplemental Table S2.

A total of 19 phyla were observed across all restroom surfaces with most sequences (≈92%) classified to one of four phyla: *Actinobacteria*, *Bacteroidetes*, *Firmicutes* or *Proteobacteria* (Figure 1A, Table S2). Previous cultivation-dependent and –independent studies have also frequently identified these as the dominant phyla in a variety of indoor environments [10]–[13]. Within these dominant phyla, taxa typically associated with human skin (e.g. *Propionibacteriaceae*, *Corynebacteriaceae*, *Staphylococcaceae* and *Streptococcaceae*) [30] were abundant on all surfaces (Figure 1A). The prevalence of skin bacteria on restroom surfaces is not surprising as most of the surfaces sampled come into direct contact with human skin, and previous studies have shown that skin associated bacteria are generally resilient and can survive on surfaces for extended periods of time [31], [32]. Many other human-associated taxa, including several lineages associated with the gut, mouth and urine, were observed on all surfaces (Figure 1A). Overall, these results demonstrate that, like other indoor environments that have been examined, the microbial communities associated with public restroom surfaces are predominantly composed of human-associated bacteria.

**Figure 2:**
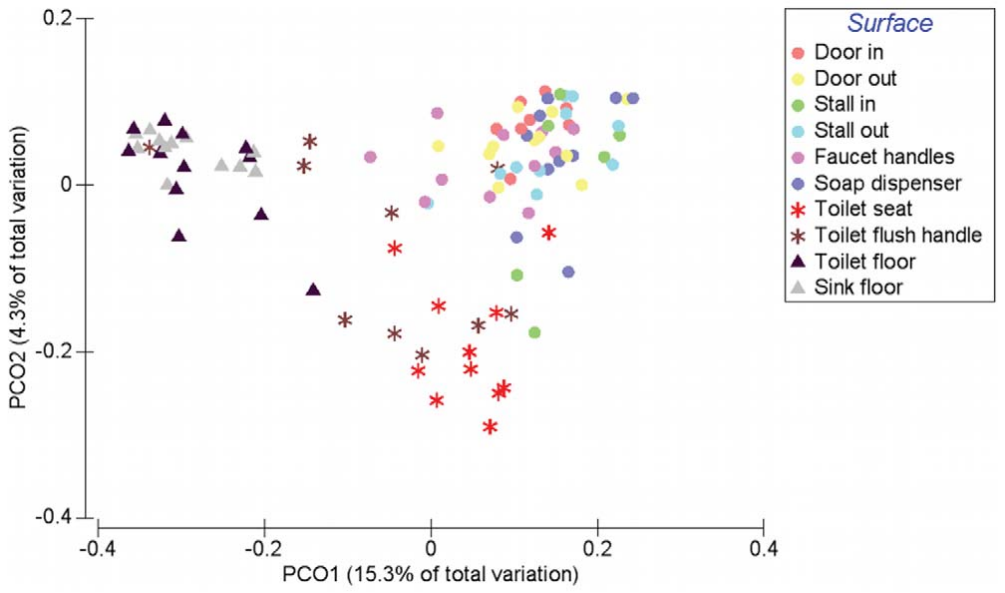
Relationship between bacterial communities associated with ten public restroom surfaces. Communities were clustered using PCoA of the unweighted UniFrac distance matrix. Each point represents a single sample. Note that the floor (triangles) and toilet (asterisks) surfaces form clusters distinct from surfaces touched with hands.

**Figure 3:**
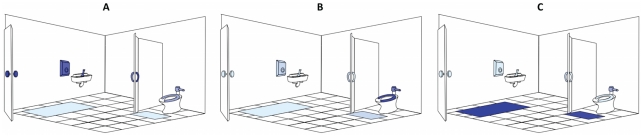
Cartoon illustrations of the relative abundance of discriminating taxa on public restroom surfaces. Light blue indicates low abundance while dark blue indicates high abundance of taxa. (**A**) Although skin-associated taxa (*Propionibacteriaceae*, *Corynebacteriaceae*, *Staphylococcaceae* and *Streptococcaceae*) were abundant on all surfaces, they were relatively more abundant on surfaces routinely touched with hands. (**B**) Gut-associated taxa (*Clostridiales*, *Clostridiales* group XI, *Ruminococcaceae*, *Lachnospiraceae*, *Prevotellaceae* and *Bacteroidaceae*) were most abundant on toilet surfaces. (**C**) Although soil-associated taxa (*Rhodobacteraceae, Rhizobiales, Microbacteriaceae* and *Nocardioidaceae*) were in low abundance on all restroom surfaces, they were relatively more abundant on the floor of the restrooms we surveyed. Figure not drawn to scale.

Comparisons of the bacterial communities on different restroom surfaces revealed that the communities clustered into three general categories: those communities found on toilet surfaces (the seat and flush handle), those communities on the restroom floor, and those communities found on surfaces routinely touched with hands (door in/out, stall in/out, faucet handles and soap dispenser) (Figure 2, Table 1). By examining the relative abundances of bacterial taxa across all of the restroom samples, we can identify taxa driving the overall community differences between these three general categories. Skin-associated bacteria dominate on those surfaces (the circles in Figure 2) that are routinely and exclusively (we hope) touched by hands and unlikely to come into direct contact with other body parts or fluids (Figure 3A). In contrast, toilet flush handles and seats (the asterisk-shaped symbols in Figure 2) were relatively enriched in *Firmicutes* (e.g. *Clostridiales*, *Ruminococcaceae*, *Lachnospiraceae*, etc.) and *Bacteroidetes* (e.g. *Prevotellaceae* and *Bacteroidaceae*) (Figure 3B). These taxa are generally associated with the human gut [26], [33]–[35] suggesting fecal contamination of these surfaces. Fecal contamination could occur either via direct contact (with feces or unclean hands) or indirectly as a toilet is flushed and water splashes or is aerosolized [36]–[38]. From a public health perspective, the high number of gut-associated taxa throughout the restrooms is concerning because enteropathogenic bacteria could be dispersed in the same way as human commensals. Floor surfaces harbored many low abundance taxa (Table S2) and were the most diverse bacterial communities, with an average of 229 OTUs per sample versus most of the other sampled locations having less than 150 OTUs per sample on average (Table S1). The high diversity of floor communities is likely due to the frequency of contact with the bottom of shoes, which would track in a diversity of microorganisms from a variety of sources including soil, which is known to be a highly-diverse microbial habitat [27], [39]. Indeed, bacteria commonly associated with soil (e.g. *Rhodobacteraceae, Rhizobiales, Microbacteriaceae* and *Nocardioidaceae*) were, on average, more abundant on floor surfaces (Figure 3C, Table S2). Interestingly, some of the toilet flush handles harbored bacterial communities similar to those found on the floor (Figure 2, Figure 3C), suggesting that some users of these toilets may operate the handle with a foot (a practice well known to germaphobes and those who have had the misfortune of using restrooms that are less than sanitary).

**Table 1:** Results of pairwise comparisons for unweighted UniFrac distances of bacterial communities associated with various surfaces of public restrooms on the University of Colorado campus using the ANOSIM test in Primer v6.

	Door in	Door out	Stall in	Stall out	Faucet handle	Soap dispenser	Toilet flush handle	Toilet seat	Toilet floor
Door in									
Door out	−0.139								
Stall in	0.149	−0.053							
Stall out	−0.074	−0.083	−0.037						
Faucet handle	−0.062	−0.011	−0.092	−0.040					
Soap dispenser	−0.020	0.014	−0.060	−0.001	0.070				
Toilet flush handle	0.376*	0.405*	0.221	0.350*	0.172*	0.470*			
Toilet seat	0.742*	0.672*	0.457*	0.586*	0.401*	0.653*	0.187*		
Toilet floor	0.995*	0.988*	0.993*	0.961*	0.758*	0.998*	0.577*	0.950*	
Sink floor	1.000*	0.995*	1.000*	0.974*	0.770*	1.000*	0.655*	0.982*	−0.033

The *R*-statistic is shown for each comparison with asterisks denoting comparisons that were statistically significant at *P*≤0.01.

While the overall community level comparisons between the communities found on the surfaces in male and female restrooms were not statistically significant (Table S3), there were gender-related differences in the relative abundances of specific taxa on some surfaces (Figure 1B, Table S2). Most notably, *Lactobacillaceae* were clearly more abundant on certain surfaces within female restrooms than male restrooms (Figure 1B). Some species of this family are the most common, and often most abundant, bacteria found in the vagina of healthy reproductive age women [40], [41] and are relatively less abundant in male urine [28], [29]. Our analysis of female urine samples collected as part of a previous study [26] (Figure 1A), found that *Lactobacillaceae* were dominant in urine, therefore implying that surfaces in the restrooms where *Lactobacillaceae* were observed were contaminated with urine. Other studies have demonstrated a similar phenomenon, with vagina-associated bacteria having also been observed in airplane restrooms [11] and a child day care facility [10]. As we found that *Lactobacillaceae* were most abundant on toilet surfaces and those touched by hands after using the toilet (with the exception of the stall in), they were likely dispersed manually after women used the toilet. Coupling these observations with those of the distribution of gut-associated bacteria indicate that routine use of toilets results in the dispersal of urine- and fecal-associated bacteria throughout the restroom. While these results are not unexpected, they do highlight the importance of hand-hygiene when using public restrooms since these surfaces could also be potential vehicles for the transmission of human pathogens. Unfortunately, previous studies have documented that college students (who are likely the most frequent users of the studied restrooms) are not always the most diligent of hand-washers [42], [43].

**Figure 4:**
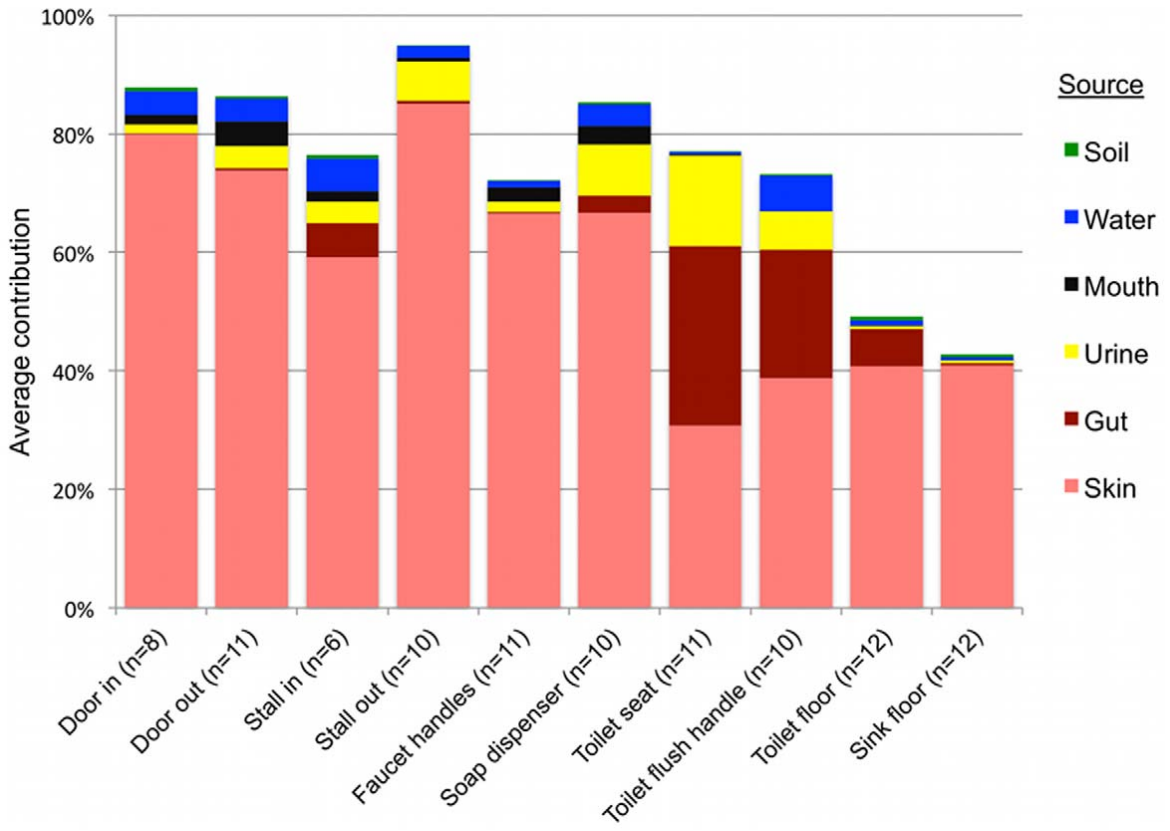
Results of SourceTracker analysis showing the average contributions of different sources to the surface-associated bacterial communities in twelve public restrooms. The “unknown” source is not shown but would bring the total of each sample up to 100%.

Results of SourceTracker analysis support the taxonomic patterns highlighted above, indicating that human skin was the primary source of bacteria on all public restroom surfaces examined, while the human gut was an important source on or around the toilet, and urine was an important source in women's restrooms (Figure 4, Table S4). Contrary to expectations (see above), soil was not identified by the SourceTracker algorithm as being a major source of bacteria on any of the surfaces, including floors (Figure 4). Although the floor samples contained family-level taxa that are common in soil, the SourceTracker algorithm probably underestimates the relative importance of sources, like soils, that contain highly diverse bacterial communities with no dominant OTUs and minimal overlap between those OTUs in the sources and those found in the surface samples. As soils typically have large numbers of OTUs that are rare (i.e. represented by very few sequences) and the OTU overlap between different soil samples is very low [27], it is difficult to identify specific OTUs indicative of a soil source. The other potential sources we examined, mouth and faucet water, made only minor bacterial contributions to restroom surface communities either because these potential source environments rarely come into contact with restroom surfaces (the mouth – we hope) or they harbor relatively low concentrations of bacteria (faucet water) (Figure 4). While we were able to identify the primary sources for most of the surfaces sampled, many other sources, such as ventilation systems or mops used by the custodial staff, could also be contributing to the restroom surface bacterial communities. More generally, the SourceTracker results demonstrate how direct comparison of bacterial communities from samples of various environment types to those gathered from other settings can be used to determine the relative contribution of that source across samples. Although many of the source-tracking results evident from the restroom surfaces sampled here are somewhat obvious, this may not always be the case in other environments or locations. We could use the same techniques to identify unexpected sources of bacteria from particular environments as was observed recently for outdoor air [44].

### Conclusion

While we have known for some time that human-associated bacteria can be readily cultivated from both domestic and public restroom surfaces, little was known about the overall composition of microbial communities associated with public restrooms or the degree to which microbes can be distributed throughout this environment by human activity. The results presented here demonstrate that human-associated bacteria dominate most public restroom surfaces and that distinct patterns of dispersal and community sources can be recognized for microbes associated with these surfaces. Although the methods used here did not provide the degree of phylogenetic resolution to directly identify likely pathogens, the prevalence of gut and skin-associated bacteria throughout the restrooms we surveyed is concerning since enteropathogens or pathogens commonly found on skin (e.g. *Staphylococcus aureus*) could readily be transmitted between individuals by the touching of restroom surfaces.

## Supporting Information

Table S1 **Public restroom surfaces sampled and comparison of alpha-diversity metrics for each restroom surface.** Note that all alpha-diversity values were determined from 500 randomly selected sequences from each sample.
(DOC)

Table S2 **Average taxonomic composition of bacterial communities associated with female (F) and male (M) public restroom surfaces.** Numbers in parentheses indicate the standard error of the mean (SEM). Taxonomy was determined using the RDP-classifier for 500 randomly selected sequences from each sample.
(DOC)

Table S3 **Results of ANOSIM test comparing the bacterial communities associated with male and female restroom surfaces.**
(DOC)

Table S4 **Results of SourceTracker analysis showing percentage of microbial community contributions of different source environments to restroom surfaces.** Values are the average of ten resamplings with the standard error of the mean reported in parentheses.
(DOC)
