# Supplementary material for: Microbial Biogeography of Public Restroom Surfaces
Source: PLoS One. 2011 Nov 23;6(11):e28132. doi: 10.1371/journal.pone.0028132 (PMC3223236; doi:10.1371/journal.pone.0028132)
Supplement: Table S1 — Public restroom surfaces sampled and comparison of alpha-diversity metrics for each restroom surface. Note that all alpha-diversity values were determined from 500 randomly selected sequences from each sample. (DOC) [file pone.0028132.s001.doc]

| Surface | Gender | Building – level | # of sequences | # of OTUs | Chao1^1^ | PD^2^ |
| --- | --- | --- | --- | --- | --- | --- |
| Door in | Female | Ekeley – 1 | 4261 | 122 | 204 | 15.40 |
| Door out | Female | Ekeley – 1 | 3756 | 146 | 220 | 13.27 |
| Stall in | Female | Ekeley – 1 | 4127 | 111 | 130 | 15.60 |
| Stall out | Female | Ekeley – 1 | 3560 | 83 | 163 | 11.24 |
| Faucet handles | Female | Ekeley – 1 | 6771 | 71 | 165 | 11.58 |
| Soap dispenser | Female | Ekeley – 1 | 3738 | 113 | 162 | 16.19 |
| Toilet seat | Female | Ekeley – 1 | 4258 | 135 | 254 | 15.34 |
| Toilet flush handle | Female | Ekeley – 1 | 4093 | 290 | 787 | 25.54 |
| Toilet floor | Female | Ekeley – 1 | 3902 | 220 | 511 | 24.06 |
| Sink floor | Female | Ekeley – 1 | 3644 | 215 | 786 | 20.18 |
| Door in | Male | Ekeley – 1 | 4942 | 123 | 152 | 15.48 |
| Door out | Male | Ekeley – 1 | -^3^ | - | - | - |
| Stall in | Male | Ekeley – 1 | - | - | - | - |
| Stall out | Male | Ekeley – 1 | 3449 | 126 | 313 | 13.55 |
| Faucet handles | Male | Ekeley – 1 | 4349 | 88 | 173 | 11.13 |
| Soap dispenser | Male | Ekeley – 1 | - | - | - | - |
| Toilet seat | Male | Ekeley – 1 | 4071 | 94 | 166 | 10.78 |
| Toilet flush handle | Male | Ekeley – 1 | 4751 | 116 | 195 | 14.71 |
| Toilet floor | Male | Ekeley – 1 | 4217 | 173 | 501 | 18.87 |
| Sink floor | Male | Ekeley – 1 | 3607 | 207 | 919 | 20.31 |
| Door in | Female | Ekeley – 2 | 3781 | 124 | 255 | 15.46 |
| Door out | Female | Ekeley – 2 | 3495 | 122 | 220 | 13.27 |
| Stall in | Female | Ekeley – 2 | - | - | - | - |
| Stall out | Female | Ekeley – 2 | 3379 | 69 | 138 | 10.88 |
| Faucet handles | Female | Ekeley – 2 | 3545 | 119 | 183 | 15.54 |
| Soap dispenser | Female | Ekeley – 2 | 1979 | 109 | 150 | 14.61 |
| Toilet seat | Female | Ekeley – 2 | 2309 | 75 | 110 | 11.75 |
| Toilet flush handle | Female | Ekeley – 2 | - | - | - | - |
| Toilet floor | Female | Ekeley – 2 | 4781 | 217 | 380 | 21.22 |
| Sink floor | Female | Ekeley – 2 | 3314 | 277 | 1061 | 24.50 |
| Door in | Male | Ekeley – 2 | - | - | - | - |
| Door out | Male | Ekeley – 2 | 3446 | 138 | 234 | 15.04 |
| Stall in | Male | Ekeley – 2 | 3675 | 108 | 163 | 15.10 |
| Stall out | Male | Ekeley – 2 | 3992 | 109 | 187 | 14.79 |
| Faucet handles | Male | Ekeley – 2 | 4190 | 100 | 222 | 13.35 |
| Soap dispenser | Male | Ekeley – 2 | 4399 | 123 | 206 | 14.77 |
| Toilet seat | Male | Ekeley – 2 | 4798 | 129 | 173 | 15.95 |
| Toilet flush handle | Male | Ekeley – 2 | 4501 | 165 | 341 | 19.88 |
| Toilet floor | Male | Ekeley – 2 | 4707 | 259 | 920 | 22.54 |
| Sink floor | Male | Ekeley – 2 | 4149 | 231 | 739 | 21.18 |
| Door in | Female | Ekeley – 3 | 3586 | 142 | 215 | 16.66 |
| Door out | Female | Ekeley – 3 | 2970 | 136 | 299 | 14.59 |
| Stall in | Female | Ekeley – 3 | - | - | - | - |
| Stall out | Female | Ekeley – 3 | 4420 | 106 | 198 | 14.84 |
| Faucet handles | Female | Ekeley – 3 | 3719 | 88 | 198 | 12.21 |
| Soap dispenser | Female | Ekeley – 3 | 2501 | 123 | 350 | 14.74 |
| Toilet seat | Female | Ekeley – 3 | 4059 | 153 | 294 | 18.09 |
| Toilet flush handle | Female | Ekeley – 3 | 3588 | 133 | 247 | 17.31 |
| Toilet floor | Female | Ekeley – 3 | 3648 | 215 | 435 | 21.99 |
| Sink floor | Female | Ekeley – 3 | 4827 | 216 | 401 | 20.95 |
| Door in | Male | Ekeley – 3 | 4110 | 108 | 157 | 15.19 |
| Door out | Male | Ekeley – 3 | 3987 | 119 | 207 | 14.71 |
| Stall in | Male | Ekeley – 3 | - | - | - | - |
| Stall out | Male | Ekeley – 3 | 3939 | 85 | 215 | 10.55 |
| Faucet handles | Male | Ekeley – 3 | 4234 | 107 | 224 | 12.15 |
| Soap dispenser | Male | Ekeley – 3 | - | - | - | - |
| Toilet seat | Male | Ekeley – 3 | 2375 | 132 | 224 | 14.21 |
| Toilet flush handle | Male | Ekeley – 3 | 3535 | 136 | 284 | 14.09 |
| Toilet floor | Male | Ekeley – 3 | 4938 | 214 | 747 | 21.33 |
| Sink floor | Male | Ekeley – 3 | 4084 | 203 | 693 | 19.41 |
| Door in | Female | Porter – 1 | 4165 | 123 | 218 | 15.47 |
| Door out | Female | Porter – 1 | 3764 | 117 | 179 | 16.82 |
| Stall in | Female | Porter – 1 | - | - | - | - |
| Stall out | Female | Porter – 1 | - | - | - | - |
| Faucet handles | Female | Porter – 1 | 3926 | 134 | 308 | 17.44 |
| Soap dispenser | Female | Porter – 1 | 5197 | 109 | 164 | 15.15 |
| Toilet seat | Female | Porter – 1 | 3919 | 125 | 177 | 14.94 |
| Toilet flush handle | Female | Porter – 1 | 4070 | 135 | 313 | 15.28 |
| Toilet floor | Female | Porter – 1 | 4209 | 237 | 716 | 22.27 |
| Sink floor | Female | Porter – 1 | 3124 | 260 | 664 | 24.90 |
| Door in | Male | Porter – 1 | - | - | - | - |
| Door out | Male | Porter – 1 | 3185 | 86 | 114 | 11.99 |
| Stall in | Male | Porter – 1 | 1597 | 165 | 279 | 17.70 |
| Stall out | Male | Porter – 1 | 513 | 88 | 165 | 11.41 |
| Faucet handles | Male | Porter – 1 | 3808 | 100 | 342 | 12.62 |
| Soap dispenser | Male | Porter – 1 | 1343 | 104 | 190 | 13.22 |
| Toilet seat | Male | Porter – 1 | 3607 | 159 | 242 | 16.50 |
| Toilet flush handle | Male | Porter – 1 | 1830 | 157 | 243 | 17.68 |
| Toilet floor | Male | Porter – 1 | 2648 | 263 | 720 | 24.39 |
| Sink floor | Male | Porter – 1 | 3385 | 256 | 879 | 25.19 |
| Door in | Female | Porter – 2 | - | - | - | - |
| Door out | Female | Porter – 2 | 3518 | 133 | 178 | 16.59 |
| Stall in | Female | Porter – 2 | - | - | - | - |
| Stall out | Female | Porter – 2 | - | - | - | - |
| Faucet handles | Female | Porter – 2 | - | - | - | - |
| Soap dispenser | Female | Porter – 2 | 1788 | 136 | 207 | 15.02 |
| Toilet seat | Female | Porter – 2 | 3544 | 97 | 174 | 11.91 |
| Toilet flush handle | Female | Porter – 2 | - | - | - | - |
| Toilet floor | Female | Porter – 2 | 2196 | 256 | 812 | 24.53 |
| Sink floor | Female | Porter – 2 | 2687 | 262 | 943 | 24.26 |
| Door in | Male | Porter – 2 | - | - | - | - |
| Door out | Male | Porter – 2 | 2120 | 106 | 160 | 13.27 |
| Stall in | Male | Porter – 2 | 2665 | 86 | 123 | 12.02 |
| Stall out | Male | Porter – 2 | 3719 | 96 | 186 | 13.50 |
| Faucet handles | Male | Porter – 2 | 3910 | 100 | 155 | 14.51 |
| Soap dispenser | Male | Porter – 2 | 4302 | 99 | 167 | 12.22 |
| Toilet seat | Male | Porter – 2 | - | - | - | - |
| Toilet flush handle | Male | Porter – 2 | 3619 | 184 | 322 | 20.57 |
| Toilet floor | Male | Porter – 2 | 3735 | 243 | 429 | 24.43 |
| Sink floor | Male | Porter – 2 | 4078 | 211 | 372 | 22.87 |
| Door in | Female | Porter – 3 | 2190 | 115 | 245 | 13.15 |
| Door out | Female | Porter – 3 | 1969 | 127 | 217 | 15.97 |
| Stall in | Female | Porter – 3 | 2001 | 136 | 231 | 15.83 |
| Stall out | Female | Porter – 3 | 2002 | 108 | 172 | 12.79 |
| Faucet handles | Female | Porter – 3 | 2331 | 30 | 43 | 4.43 |
| Soap dispenser | Female | Porter – 3 | 2009 | 136 | 199 | 15.97 |
| Toilet seat | Female | Porter – 3 | 2295 | 118 | 367 | 13.16 |
| Toilet flush handle | Female | Porter – 3 | 1997 | 197 | 502 | 21.97 |
| Toilet floor | Female | Porter – 3 | 2443 | 225 | 625 | 21.86 |
| Sink floor | Female | Porter – 3 | 1347 | 232 | 642 | 23.38 |
| Door in | Male | Porter – 3 | 2143 | 108 | 250 | 14.14 |
| Door out | Male | Porter – 3 | 1069 | 131 | 254 | 14.64 |
| Stall in | Male | Porter – 3 | 1982 | 154 | 301 | 17.00 |
| Stall out | Male | Porter – 3 | 2290 | 102 | 322 | 13.23 |
| Faucet handles | Male | Porter – 3 | 2824 | 55 | 154 | 7.99 |
| Soap dispenser | Male | Porter – 3 | 1565 | 93 | 107 | 12.88 |
| Toilet seat | Male | Porter – 3 | 1363 | 156 | 233 | 17.42 |
| Toilet flush handle | Male | Porter – 3 | 2362 | 136 | 193 | 16.65 |
| Toilet floor | Male | Porter – 3 | 2260 | 176 | 352 | 21.12 |
| Sink floor | Male | Porter – 3 | 2264 | 220 | 526 | 23.77 |
| Water | Male | Ekeley – 1 | 2744 | 30 | 35 | 5.51 |
| Water | Male | Ekeley – 2 | 2161 | 25 | 36 | 5.03 |
| Water | Male | Ekeley – 3 | 3178 | 26 | 27 | 4.63 |
| Water | Male | Porter – 1 | - | - | - | - |
| Water | Male | Porter – 2 | 5084 | 24 | 35 | 4.60 |
| Water | Male | Porter – 3 | 3249 | 28 | 39 | 5.11 |

^1^Rounded to nearest whole number

^2^Faith’s Phylogenetic Diversity

^3^Denotes samples for which at least 500 quality sequences were not obtained
