# Supplementary material for: Microbial Biogeography of Public Restroom Surfaces
Source: PLoS One. 2011 Nov 23;6(11):e28132. doi: 10.1371/journal.pone.0028132 (PMC3223236; doi:10.1371/journal.pone.0028132)
Supplement: Table S2 — Average taxonomic composition of bacterial communities associated with female (F) and male (M) public restroom surfaces. Numbers in parentheses indicate the standard error of the mean (SEM). Taxonomy was determined using the RDP-classifier for 500 randomly selected sequences from each sample. (DOC) [file pone.0028132.s002.doc]

|  | Door in | | Door out | | Stall in | | Stall out | | Faucet handles | | Soap dispenser | | Toilet seat | | Toilet flush handle | | Toilet floor | | Sink floor | |
| --- | --- | --- | --- | --- | --- | --- | --- | --- | --- | --- | --- | --- | --- | --- | --- | --- | --- | --- | --- | --- |
| Taxa | F  n=5 | M  n=3 | F  n=6 | M  n=5 | F  n=2 | M  n=4 | F  n=4 | M  n=6 | F  n=5 | M  n=6 | F  n=6 | M  n=4 | F  n=6 | M  n=5 | F  n=4 | M  n=6 | F  n=6 | M  n=6 | F  n=6 | M  n=6 |
| *Actinobacteria* |  |  |  |  |  |  |  |  |  |  |  |  |  |  |  |  |  |  |  |  |
| *Propionibacteriaceae* | 37.48  (3.35) | 37.67  (2.27) | 17.47  (2.45) | 25.76  (4.02) | 20.20  (4.60) | 17.05  (6.30) | 32.60  (4.36) | 36.97  (3.90) | 9.48  (2.90) | 28.87  (8.75) | 21.43  (4.07) | 28.95  (8.57) | 1.70  (0.53) | 4.08  (0.89) | 5.15  (1.39) | 12.27  (4.13) | 5.53  (0.58) | 5.53  (0.67) | 6.03  (0.62) | 5.23  (0.41) |
| *Corynebacteriaceae* | 2.76  (0.58) | 5.87  (1.12) | 4.10  (0.90) | 7.20  (0.84) | 3.50  (0.30) | 6.80  (2.88) | 2.15  (0.29) | 7.50  (2.10) | 2.52  (1.38) | 15.13  (6.99) | 3.47  (1.31) | 5.65  (1.44) | 8.53  (3.42) | 27.44  (5.91) | 7.00  (4.69) | 10.20  (3.74) | 1.73  (0.56) | 1.90  (0.42) | 1.37  (0.31) | 1.73  (0.21) |
| *Micrococcaceae* | 2.24  (0.39) | 2.27  (024) | 2.33  (0.44) | 3.48  (0.37) | 3.30  (1.30) | 3.90  (1.20) | 2.05  (0.44) | 2.00  (0.46) | 3.04  (1.02) | 9.17  (6.68) | 2.07  (0.53) | 3.45  (0.71) | 2.43  (1.04) | 0.56  (0.25) | 2.60  (1.37) | 2.07  (0.84) | 8.83  (0.84) | 9.23  (1.41) | 11.80  (0.96) | 10.23  (1.89) |
| *Actinomycetales* | 1.64  (0.28) | 1.60  (0.12) | 1.43  (0.36) | 1.04  (0.19) | 0.70  (0.10) | 0.95  (0.30) | 0.90  (0.21) | 2.20  (0.46) | 0.84  (0.16) | 2.23  (0.55) | 0.90  (0.29) | 1.35  (0.69) | 0.37  (0.12) | 0.56  (0.20) | 2.55  (0.88) | 1.27  (0.63) | 5.03  (0.42) | 3.83  (0.88) | 5.33  (0.73) | 4.07  (0.54) |
| *Actinomycetaceae* | 1.20  (0.24) | 1.33  (0.57) | 5.57  (3.06) | 2.12  (0.22) | 1.40  (0.40) | 1.30  (0.49) | 1.25  (0.36) | 0.77  (0.34) | 0.92  (0.31) | 0.77  (0.24) | 1.63  (0.52) | 1.20  (0.12) | 0.60  (0.23) | 0.28  (0.14) | 0.50  (0.24) | 0.43  (0.17) | 0.10  (0.04) | 0.13  (0.04) | 0.07  (0.04) | 0.10  (0.04) |
| *Microbacteriaceae* | 0.20  (0.11) | 0.33  (0.18) | 0.33  (0.13) | 0.36  (0.10) | 0.90  (0.50) | 0.45  (0.13) | 0.35  (0.22) | 0.23  (0.11) | 0.20  (0.15) | 0.73  (0.34) | 0.30  (0.19) | 0.50  (0.19) | 0.33  (0.13) | 0.28  (0.28) | 1.80  (0.70) | 0.63  (0.13) | 2.73  (0.37) | 2.30  (0.56) | 2.73  (0.37) | 3.27  (0.82) |
| *Nocardioidaceae* | 0.20  (0.11) | 0.07  (0.07) | 0.10  (0.07) | 0.12  (0.05) | 0.20  (0.20) | 0.15  (0.15) | 0.05  (0.05) | 0.17  (0.11) | 0.08  (0.08) | 0.17  (0.08) | 0.30  (0.11) | 0.10  (0.06) | 0.17  (0.10) | 0.12  (0.08) | 2.00  (1.22) | 0.37  (0.12) | 2.30  (0.31) | 2.17  (0.55) | 2.77  (0.34) | 2.30  (0.49) |
| Other | 2.00  (0.68) | 2.13  (0.35) | 1.23  (0.50) | 1.36  (0.37) | 2.70  (0.50) | 2.25  (0.40) | 0.75  (0.45) | 1.03  (0.48) | 10.84  (9.01) | 3.07  (1.96) | 1.67  (0.44) | 0.75  (0.10) | 0.77  (0.28) | 1.48  (0.36) | 6.60  (3.61) | 3.10  (0.65) | 7.50  (0.76) | 7.70  (1.41) | 8.17  (0.57) | 8.17  (1.25) |
| *Bacteriodetes* |  |  |  |  |  |  |  |  |  |  |  |  |  |  |  |  |  |  |  |  |
| *Prevotellaceae* | 1.72  (0.89) | 0.40  (0.31) | 1.50  (0.95) | 0.52  (0.38) | 1.00  (0.40) | 0.60  (0.18) | 1.10  (0.37) | 0.20  (0.05) | 0.48  (0.20) | 0.63  (0.28) | 3.13  (1.58) | 0.60  (0.14) | 1.47  (0.56) | 3.40  (1.73) | 0.70  (0.51) | 7.90  (4.45) | 0.57  (0.41) | 0.33  (0.11) | 0.37  (0.17) | 0.40  (0.20) |
| *Bacteroidaceae* | 0.08  (0.08) | - | 0.13  (0.08) | 0.12  (0.08) | - | 2.15  (1.65) | 0.05  (0.05) | 0.17  (0.11) | 0.32  (0.14) | 0.07  (0.04) | 0.87  (0.71) | 0.05  (0.05) | 7.97  (4.20) | 2.64  (0.70) | 1.00  (0.93) | 3.80  (1.30) | 0.43  (0.28) | 2.83  (2.23) | 0.23  (0.12) | 0.20  (0.16) |
| *Flavobacteriaceae* | 0.68  (0.19) | 0.80  (0.46) | 0.97  (0.19) | 0.24  (0.07) | - | 0.90  (0.24) | 0.35  (0.13) | 0.50  (0.18) | 0.16  (0.12) | 0.57  (0.42) | 0.37  (0.11) | 0.30  (0.13) | 0.33  (0.16) | 0.32  (0.16) | 1.25  (0.45) | 0.53  (0.19) | 1.93  (0.21) | 1.43  (0.45) | 1.50  (0.36) | 3.00  (0.61) |
| *Cytophagaceae* | 0.24  (0.07) | 0.13  (0.13) | 0.17  (0.06) | 0.08  (0.05) | 0.20  (0.20) | - | 0.10  (0.06) | 0.20  (0.09) | 0.04  (0.04) | 0.07  (0.04) | 0.30  (0.19) | - | 0.10  (0.07) | 0.04  (0.04) | 0.80  (0.28) | 1.00  (0.57) | 2.07  (0.27) | 1.80  (0.31) | 2.77  (0.29) | 2.07  (0.29) |
| *Chitinophagaceae* | 0.60  (0.26) | 0.93  (0.29) | 0.53  (0.26) | 0.28  (0.20) | 1.30  (0.50) | 0.20  (0.00) | 0.25  (0.15) | 0.30  (0.12) | 0.32  (0.16) | 0.10  (0.10) | 0.50  (0.20) | 0.45  (0.15) | 0.30  (0.15) | 0.28  (0.23) | 0.65  (0.29) | 0.60  (0.29) | 0.50  (0.21) | 0.13  (0.10) | 0.50  (0.15) | 0.47  (0.04) |
| Other | 0.84  (0.40) | 0.73  (0.13) | 1.00  (0.20) | 0.80  (0.14) | 2.40  (0.40) | 2.10  (1.08) | 0.30  (0.13) | 0.67  (0.08) | 0.60  (0.23) | 0.83  (0.17) | 0.93  (0.35) | 1.05  (0.15) | 0.77  (0.20) | 1.56  (0.33) | 1.35  (0.28) | 1.10  (0.34) | 1.10  (0.09) | 1.40  (0.52) | 1.03  (0.17) | 1.40  (0.29) |
| *Firmicutes* |  |  |  |  |  |  |  |  |  |  |  |  |  |  |  |  |  |  |  |  |
| *Lactobacillaceae* | 0.80  (0.20) | 0.27  (0.27) | 4.20  (1.90) | 0.36  (0.07) | 5.60  (1.60) | 0.05  (0.05) | 12.90  (11.91) | 0.33  (0.26) | 2.60  (0.93) | 1.27  (0.82) | 7.07  (4.17) | 0.45  (0.22) | 20.27  (10.28) | 0.48  (0.26) | 8.80  (4.89) | 0.67  (0.29) | 1.03  (0.38) | 0.47  (0.14) | 0.70  (0.22) | 0.43  (0.20) |
| *Streptococcaceae* | 7.88  (0.81) | 4.87  (0.24) | 7.97  (1.38) | 7.44  (1.07) | 8.80  (1.00) | 3.90  (1.08) | 7.35  (2.35) | 6.77  (2.78) | 2.76  (0.77) | 4.33  (1.49) | 6.97  (2.01) | 5.05  (1.68) | 1.20  (0.45) | 0.76  (0.13) | 1.65  (0.66) | 1.60  (0.53) | 1.67  (0.59) | 1.13  (0.48) | 1.17  (0.40) | 1.03  (0.17) |
| *Staphylococcaceae* | 6.72  (0.77) | 6.93  (0.77) | 13.27  (3.45) | 15.00  (2.23) | 5.00  (2.20) | 3.70  (1.40) | 7.85  (2.88) | 6.23  (1.09) | 2.36  (0.83) | 4.80  (1.25) | 3.33  (0.63) | 4.55  (1.51) | 2.37  (1.00) | 2.24  (0.54) | 5.55  (4.00) | 2.93  (0.82) | 1.00  (0.25) | 1.17  (0.38) | 0.60  (0.07) | 0.87  (0.20) |
| *Clostridiales Gp XI* | 1.20  (0.22) | 1.40  (0.50) | 1.23  (0.39) | 1.64  (0.31) | 2.20  (1.20) | 1.65  (1.07) | 0.30  (0.13) | 1.20  (0.61) | 1.12  (0.56) | 0.73  (0.35) | 3.40  (1.64) | 0.55  (0.19) | 3.03  (1.15) | 14.92  (5.04) | 2.85  (1.59) | 3.50  (1.95) | 0.57  (0.16) | 1.07  (0.59) | 0.40  (0.12) | 0.33  (0.10) |
| *Clostridiales* | 0.28  (0.23) | 0.20  (0.12) | 0.47  (0.20) | 0.84  (0.37) | 1.00  (0.40) | 2.40  (1.39) | 0.20  (0.08) | 0.47  (0.26) | 0.24  (0.12) | 0.43  (0.21) | 1.60  (0.91) | 0.25  (0.13) | 6.93  (3.57) | 8.04  (2.33) | 1.45  (1.19) | 6.53  (2.75) | 0.50  (0.24) | 2.63  (1.47) | 0.73  (0.28) | 0.57  (0.20) |
| *Ruminococcaceae* | 0.12  (0.08) | 0.20  (0.20) | 0.30  (0.07) | 0.32  (0.27) | 0.20  (0.20) | 2.10  (1.17) | 0.10  (0.10) | 0.47  (0.18) | 0.56  (0.32) | 0.10  (0.07) | 2.37  (2.05) | 0.15  (0.10) | 4.83  (1.66) | 7.84  (2.17) | 1.40  (1.33) | 6.23  (2.31) | 1.00  (0.29) | 4.70  (2.93) | 1.10  (0.55) | 0.50  (0.25) |
| *Lachnospiraceae* | 0.12  (0.08) | - | 1.00  (0.56) | 0.60  (0.24) | 1.10  (0.70) | 2.55  (1.53) | 0.25  (0.10) | 0.73  (0.27) | 0.44  (0.19) | 0.17  (0.11) | 1.27  (0.89) | 0.25  (0.15) | 7.47  (3.64) | 7.24  (2.16) | 1.30  (1.10) | 5.53  (1.92) | 0.40  (0.20) | 2.40  (0.99) | 0.60  (0.29) | 0.23  (0.13) |
| *Bacillaceae* | 0.28  (0.10) | 4.33  (4.03) | 0.03  (0.03) | 0.60  (0.50) | 0.30  (0.10) | 0.05  (0.05) | 0.20  (0.08) | 0.20  (0.13) | - | 0.03  (0.03) | 0.10  (0.04) | 14.10  (14.10) | 0.03  (0.03) | 0.04  (0.04) | 0.05  (0.05) | 0.07  (0.04) | 0.07  (0.07) | 0.10  (0.07) | 0.10  (0.07) | 0.20  (0.10) |
| *Veillonellaceae* | 1.44  (0.25) | 0.67  (0.24) | 2.37  (1.03) | 0.56  (0.20) | 0.50  (0.10) | 1.00  (0.27) | 0.55  (0.17) | 0.30  (0.15) | 0.40  (0.21) | 0.50  (0.25) | 1.10  (0.18) | 0.65  (0.39) | 0.67  (0.16) | 0.60  (0.38) | 0.20  (0.08) | 0.43  (0.15) | 0.07  (0.04) | 0.63  (0.48) | 0.10  (0.07) | 0.07  (0.04) |
| Other | 1.40  (0.20) | 0.80  (0.31) | 1.23  (0.20) | 2.40  (0.62) | 1.30  (0.10) | 3.25  (1.12) | 1.00  (0.48) | 1.63  (0.37) | 4.00  (2.92) | 1.27  (0.31) | 2.43  (0.68) | 1.00  (0.44) | 3.77  (1.59) | 4.92  (0.96) | 1.90  (0.93) | 4.67  (1.77) | 1.53  (0.18) | 1.73  (0.47) | 1.07  (0.30) | 1.47  (0.42) |
| *Proteobacteria* |  |  |  |  |  |  |  |  |  |  |  |  |  |  |  |  |  |  |  |  |
| *Alphaproteobacteria* |  |  |  |  |  |  |  |  |  |  |  |  |  |  |  |  |  |  |  |  |
| *Sphingomonadaceae* | 1.44  (0.38) | 0.53  (0.13) | 0.83  (0.20) | 1.48  (0.52) | 2.10  (0.50) | 2.20  (0.34) | 0.70  (0.19) | 0.83  (0.38) | 14.44  (5.97) | 0.67  (0.14) | 1.73  (0.30) | 1.10  (0.34) | 1.93  (1.19) | 0.48  (0.38) | 7.70  (2.34) | 4.07  (2.79) | 3.87  (0.58) | 3.27  (0.51) | 4.50  (0.34) | 3.40  (0.33) |
| *Rhizobiales* | 0.28  (0.15) | 0.60  (0.23) | 0.30  (0.18) | 0.08  (0.05) | 0.60  (0.40) | 0.40  (0.12) | 0.10  (0.06) | 0.20  (0.07) | 0.24  (0.19) | 0.23  (0.06) | 0.40  (0.14) | 0.30  (0.17) | 0.50  (0.22) | 0.12  (0.05) | 1.15  (0.46) | 0.30  (0.14) | 1.10  (0.26) | 0.93  (0.10) | 1.07  (0.41) | 1.07  (0.22) |
| *Caulobacteraceae* | 0.84  (0.26) | 0.53  (0.18) | 0.73  (0.35) | 0.64  (0.25) | 0.80  (0.60) | 9.20  (5.26) | 0.15  (0.15) | 4.57  (2.70) | 2.20  (1.18) | 1.30  (0.54) | 1.87  (1.06) | 4.55  (3.23) | 1.83  (1.64) | 1.28  (0.65) | 0.65  (0.34) | 1.63  (0.55) | 0.67  (0.20) | 0.73  (0.18) | 0.70  (0.19) | 0.97  (0.20) |
| *Rhodobacteraceae* | 0.36  (0.13) | 0.53  (0.13) | 0.93  (0.38) | 0.48  (0.26) | 0.40  (0.20) | 2.10  (1.22) | 0.25  (0.10) | 1.30  (0.65) | 0.84  (0.74) | 0.90  (0.45) | 0.70  (0.42) | 0.85  (0.26) | 0.17  (0.06) | 0.40  (0.23) | 2.55  (1.11) | 0.67  (0.36) | 4.30  (0.74) | 3.33  (0.55) | 4.63  (1.06) | 3.33  (0.71) |
| *Acetobacteraceae* | 0.32  (0.14) | 0.33  (0.07) | 0.97  (0.52) | 0.40  (0.19) | 0.30  (0.30) | 2.05  (0.87) | 0.15  (0.10) | 0.43  (0.29) | 3.36  (1.64) | 0.83  (0.39) | 0.97  (0.40) | 0.40  (0.34) | 0.63  (0.26) | 0.08  (0.05) | 0.60  (0.36) | 0.27  (0.11) | 1.43  (0.32) | 1.60  (0.36) | 1.83  (0.34) | 2.53  (0.22) |
| *Bradyrhizobiaceae* | 1.08  (0.28) | 0.80  (0.23) | 0.50  (0.32) | 0.96  (0.42) | 1.70  (1.10) | 1.05  (0.40) | 0.70  (0.31) | 0.33  (0.12) | 0.24  (0.12) | 0.23  (0.10) | 1.47  (0.36) | 1.30  (0.39) | 0.53  (0.19) | 0.48  (0.29) | 0.50  (0.17) | 1.17  (0.41) | 0.73  (0.21) | 0.37  (0.12) | 0.57  (0.08) | 1.03  (0.27) |
| Other | 1.48  (0.33) | 1.47  (0.29) | 1.47  (0.37) | 1.80  (0.64) | 3.50  (0.70) | 1.95  (0.84) | 0.80  (0.36) | 1.83  (0.68) | 2.48  (0.79) | 3.50  (2.79) | 1.73  (0.43) | 1.70  (0.51) | 1.47  (0.84) | 0.60  (0.20) | 2.55  (1.53) | 1.70  (0.38) | 3.53  (0.57) | 3.03  (0.75) | 3.97  (0.40) | 4.07  (0.63) |
| *Betaproteobacteria* |  |  |  |  |  |  |  |  |  |  |  |  |  |  |  |  |  |  |  |  |
| *Burkholderiales* | 2.20  (0.99) | 4.73  (2.44) | 2.23  (1.02) | 3.24  (1.54) | 3.80  (1.40) | 4.10  (1.20) | 1.20  (0.14) | 1.50  (0.88) | 0.40  (0.26) | 0.83  (0.34) | 1.33  (0.26) | 2.35  (0.59) | 0.93  (0.37) | 1.28  (0.36) | 1.00  (0.08) | 1.43  (0.23) | 0.97  (0.34) | 0.70  (0.29) | 0.30  (0.09) | 0.43  (0.10) |
| *Comamonadaceae* | 1.64  (0.64) | 1.73  (0.98) | 1.73  (0.70) | 1.40  (0.56) | 2.50  (2.10) | 2.50  (0.97) | 0.75  (0.10) | 2.00  (0.54) | 0.32  (0.08) | 0.80  (0.49) | 1.63  (0.28) | 1.30  (0.38) | 2.03  (0.95) | 0.64  (0.16) | 1.90  (0.62) | 1.20  (0.36) | 1.90  (0.25) | 2.07  (0.53) | 2.03  (0.28) | 1.77  (0.42) |
| *Oxalobacteraceae* | 0.24  (0.10) | 0.33  (0.33) | 0.60  (0.23) | 0.56  (0.19) | 0.90  (0.90) | 0.10  (0.10) | 0.40  (0.16) | 0.07  (0.04) | 0.28  (0.10) | 0.03  (0.03) | 0.77  (0.34) | 0.45  (0.22) | 0.07  (0.04) | 0.16  (0.10) | 0.75  (0.28) | 1.07  (0.57) | 3.03  (0.48) | 1.87  (0.23) | 2.87  (0.43) | 2.43  (0.21) |
| Other | 1.96  (0.41) | 0.80  (0.20) | 1.30  (0.33) | 1.20  (0.27) | 2.90  (1.70) | 1.30  (0.37) | 1.00  (0.14) | 0.73  (0.13) | 1.28  (0.71) | 1.07  (0.47) | 1.97  (0.35) | 1.30  (0.62) | 2.10  (1.07) | 0.60  (0.22) | 0.70  (0.17) | 0.57  (0.18) | 0.57  (0.14) | 0.60  (0.13) | 0.53  (0.13) | 0.40  (0.12) |
| *Deltaproteobacteria* | - | - | 0.03  (0.03) | 0.32  (0.27) | - | 0.05  (0.05) | - | 0.03  (0.03) | 0.12  (0.08) | - | 0.17  (0.08) | 0.05  (0.05) | 0.27  (0.13) | - | 0.15  (0.15) | 0.07  (0.04) | 0.33  (0.08) | 0.23  (0.10) | 0.13  (0.07) | 0.10  (0.10) |
| *Epsilonproteobacteria* | 0.12  (0.08) | - | 0.33  (0.22) | 0.24  (0.24) | 0.10  (0.10) | - | 0.05  (0.05) | - | 0.08  (0.05) | 0.13  (0.07) | 0.07  (0.04) | - | 0.17  (0.17) | 0.04  (0.04) | - | 0.03  (0.03) | 0.10  (0.10) | - | - | - |
| *Gammaproteobacteria* |  |  |  |  |  |  |  |  |  |  |  |  |  |  |  |  |  |  |  |  |
| *Moraxellaceae* | 1.52  (0.51) | 2.80  (0.12) | 5.17  (2.89) | 3.20  (1.45) | 2.70  (0.50) | 4.55  (1.28) | 1.15  (0.34) | 4.13  (1.28) | 19.04  (8.79) | 5.10  (2.81) | 3.73  (0.73) | 3.10  (2.05) | 3.70  (1.82) | 0.36  (0.15) | 1.10  (0.56) | 0.97  (0.14) | 9.77  (3.75) | 11.13  (5.49) | 7.10  (2.92) | 11.47  (4.90) |
| *Pseudomonadaceae* | 1.36  (0.31) | 1.80  (0.12) | 1.50  (0.43) | 1.04  (0.16) | 3.30  (0.30) | 1.00  (0.45) | 0.60  (0.08) | 0.63  (0.14) | 1.36  (0.88) | 1.40  (1.07) | 1.73  (0.83) | 1.15  (0.51) | 2.40  (2.05) | 0.24  (0.12) | 1.35  (0.76) | 0.60  (0.09) | 1.50  (0.25) | 0.83  (0.24) | 1.50  (0.26) | 2.67  (1.14) |
| *Enterobacteriaceae* | 0.96  (0.45) | 1.00  (0.20) | 0.90  (0.70) | 0.44  (0.17) | 2.00  (1.60) | 0.45  (0.39) | 3.50  (2.53) | 0.57  (0.27) | 0.12  (0.05) | 0.97  (0.78) | 1.17  (0.39) | 1.05  (0.46) | 0.33  (0.13) | 0.24  (0.24) | 5.40  (4.05) | 0.27  (0.16) | 1.60  (0.39) | 1.00  (0.54) | 1.60  (0.76) | 0.73  (0.39) |
| *Pasteurellaceae* | 1.32  (0.36) | 0.80  (0.12) | 0.83  (0.22) | 1.48  (0.32) | 0.60  (0.40) | 0.70  (0.37) | 1.15  (0.50) | 0.90  (0.26) | 0.96  (0.79) | 0.87  (0.23) | 0.60  (0.19) | 0.85  (0.26) | 0.30  (0.15) | 0.20  (0.13) | 0.20  (0.14) | 0.20  (0.09) | 0.20  (0.07) | 0.07  (0.04) | 0.13  (0.07) | 0.10  (0.07) |
| *Xanthomonadaceae* | 0.76  (0.26) | 0.60  (0.50) | 0.27  (0.04) | 0.32  (0.14) | 0.40  (0.20) | 0.60  (0.14) | 0.40  (0.08) | 0.13  (0.07) | 0.12  (0.05) | 0.23  (0.13) | 0.33  (0.13) | 0.15  (0.10) | 0.67  (0.59) | 0.12  (0.12) | 1.10  (0.90) | 0.20  (0.10) | 0.97  (0.31) | 0.37  (0.12) | 0.77  (0.25) | 1.07  (0.42) |
| Other | 0.44  (0.15) | 0.07  (0.07) | 0.10  (0.10) | 0.32  (0.19) | 0.50  (0.30) | 0.10  (0.10) | 0.30  (0.24) | - | 0.20  (0.13) | 0.07  (0.07) | 0.13  (0.07) | 0.15  (0.10) | 0.13  (0.10) | - | 1.70  (0.56) | 0.13  (0.07) | 0.43  (0.15) | 0.37  (0.15) | 0.23  (0.12) | 0.63  (0.14) |
| Unclassified | 1.00  (0.36) | 2.80  (1.70) | 0.70  (0.43) | 1.56  (0.55) | 1.40  (0.20) | 1.30  (0.37) | 0.25  (0.13) | 0.37  (0.17) | 0.24  (0.10) | 0.73  (0.42) | 0.47  (0.11) | 0.70  (0.31) | 0.40  (0.12) | 0.20  (0.09) | 0.90  (0.31) | 1.00  (0.30) | 1.27  (0.30) | 0.67  (0.16) | 0.83  (0.12) | 0.47  (0.17) |
| *Acidobacteria* | 0.40  (0.35) | 0.13  (0.13) | 0.47  (0.43) | 0.08  (0.05) | 0.10  (0.10) | - | 0.10  (0.06) | 0.20  (0.07) | 0.08  (0.08) | 0.03  (0.03) | 0.03  (0.03) | 0.10  (0.10) | 0.13  (0.07) | 0.08  (0.05) | 1.05  (0.53) | 0.17  (0.13) | 0.37  (0.15) | 0.40  (0.09) | 0.80  (0.12) | 0.47  (0.14) |
| *Chloroflexi* | - | - | - | - | - | - | - | - | - | - | - | - | - | - | 0.25  (0.25) | - | - | 0.07  (0.04) | - | 0.10  (0.05) |
| *Cyanobacteria* | 5.24  (1.16) | 2.93  (0.33) | 4.77  (1.19) | 2.36  (0.56) | 2.30  (0.50) | 1.60  (0.48) | 11.80  (8.95) | 6.63  (4.58) | 4.96  (2.71) | 1.77  (0.74) | 6.10  (1.72) | 3.55  (1.31) | 1.63  (0.53) | 0.76  (0.47) | 2.00  (1.61) | 2.23  (1.10) | 8.57  (2.87) | 5.43  (0.78) | 7.67  (1.85) | 7.70  (1.63) |
| *Deinococcus-Thermus* | 0.32  (0.16) | 0.20  (0.12) | 0.27  (0.11) | 0.20  (0.09) | 0.20  (0.20) | 0.05  (0.05) | - | - | 0.60  (0.50) | 0.30  (0.23) | 0.10  (0.07) | 0.20  (0.12) | 0.03  (0.03) | 0.04  (0.04) | 0.05  (0.05) | 0.33  (0.33) | 0.83  (0.12) | 0.97  (0.39) | 1.07  (0.25) | 0.60  (0.12) |
| *Fusobacteria* | 0.68  (0.27) | - | 2.23  (1.36) | 0.72  (0.23) | 0.10  (0.10) | 0.55  (0.22) | 0.60  (0.14) | 0.37  (0.22) | 0.68  (0.38) | 0.63  (0.16) | 0.73  (0.35) | 0.30  (0.17) | 0.10  (0.07) | 0.08  (0.08) | 0.20  (0.20) | 0.27  (0.14) | 0.03  (0.03) | - | 0.07  (0.07) | 0.03  (0.03) |
| *Gemmatimonadetes* | - | - | - | - | 0.10  (0.10) | - | - | - | - | - | - | - | - | - | 0.05  (0.05) | 0.07  (0.04) | 0.13  (0.10) | 0.03  (0.03) | - | - |
| *OD1* | - | - | - | - | - | - | - | - | - | 0.03  (0.03) | - | - | - | - | 0.05  (0.05) | - | 0.03  (0.03) | - | - | - |
| *OP11* | - | - | - | - | - | - | - | - | - | - | - | - | - | - | - | - | - | - | 0.03  (0.03) | - |
| *Planctomycetes* | - | - | - | - | - | - | - | - | - | - | - | - | - | 0.04  (0.04) | - | - | - | - | - | 0.07  (0.04) |
| *Spirochaetes* | - | - | - | 0.04  (0.04) | - | 0.05  (0.05) | - | - | - | - | - | - | - | - | - | - | 0.03  (0.03) | - | - | - |
| *SR1* | - | - | - | - | - | - | - | - | - | - | - | - | 0.07  (0.07) | - | - | - | - | - | - | - |
| *Synergistetes* | - | - | - | - | - | - | - | - | - | - | - | - | 0.03  (0.03) | - | 0.05  (0.05) | - | - | 0.03  (0.03) | - | - |
| *Tenercutes* | - | - | - | - | - | - | - | - | - | 0.03  (0.03) | 0.27  (0.27) | - | 0.07  (0.07) | - | - | 0.07  (0.07) | - | - | - | - |
| *TM7* | 0.04  (0.04) | 0.13  (0.13) | 0.47  (0.39) | 0.20  (0.11) | 0.10  (0.10) | 0.05  (0.05) | 0.05  (0.05) | - | 0.12  (0.12) | 0.03  (0.03) | 0.20  (0.07) | - | - | 0.16  (0.12) | 0.55  (0.42) | 0.23  (0.15) | 0.13  (0.08) | 0.40  (0.19) | 0.33  (0.10) | 0.70  (0.43) |
| *Verrucomicrobia* | 0.04  (0.04) | - | - | - | - | - | - | 0.03  (0.03) | 0.04  (0.04) | - | - | - | - | - | 0.15  (0.10) | 0.03  (0.03) | 0.07  (0.07) | 0.13  (0.10) | 0.07  (0.04) | - |
| *Bacteria^!^* | 1.84  (0.53) | 0.40  (0.12) | 1.43  (0.38) | 1.84  (0.82) | 2.80  (2.60) | 2.35  (0.59) | 0.90  (0.37) | 0.97  (0.26) | 0.88  (0.20) | 1.20  (0.35) | 2.07  (0.93) | 1.55  (0.49) | 0.97  (0.26) | 1.20  (0.32) | 4.55  (3.16) | 1.63  (0.30) | 3.33  (0.25) | 2.60  (0.51) | 3.40  (0.45) | 3.33  (0.37) |
| Unclassified | - | - | - | 0.16  (0.10) | - | 0.20  (0.20) | - | - | - | 0.03  (0.03) | 0.03  (0.03) | 0.10  (0.10) | - | - | - | - | - | - | 0.03  (0.03) | - |
