# Supplementary material for: Microbial Biogeography of Public Restroom Surfaces
Source: PLoS One. 2011 Nov 23;6(11):e28132. doi: 10.1371/journal.pone.0028132 (PMC3223236; doi:10.1371/journal.pone.0028132)
Supplement: Table S3 — Results of ANOSIM test comparing the bacterial communities associated with male and female restroom surfaces. (DOC) [file pone.0028132.s003.doc]

|  | *R*-statistic | *P*-value |  |
| --- | --- | --- | --- |
| All surfaces – global | 0.030 | 0.039 | No difference between male/female |
| Door in | 0.477 | 0.036 | No difference between male/female |
| Door out | 0.144 | 0.106 | No difference between male/female |
| Stall in | -0.036 | 0.533 | No difference between male/female |
| Stall out | 0.016 | 0.433 | No difference between male/female |
| Faucet handle | 0.069 | 0.188 | No difference between male/female |
| Soap dispenser | 0.099 | 0.271 | No difference between male/female |
| Toilet seat | 0.176 | 0.050 | No difference between male/female |
| Toilet flush handle | 0.119 | 0.190 | No difference between male/female |
| Toilet floor | -0.006 | 0.459 | No difference between male/female |
| Sink floor | -0.056 | 0.706 | No difference between male/female |
