# Supplementary material for: Microbial Biogeography of Public Restroom Surfaces
Source: PLoS One. 2011 Nov 23;6(11):e28132. doi: 10.1371/journal.pone.0028132 (PMC3223236; doi:10.1371/journal.pone.0028132)
Supplement: Table S4 — Results of SourceTracker analysis showing percentage of microbial community contributions of different source environments to restroom surfaces. Values are the average of ten resamplings with the standard error of the mean reported in parentheses. (DOC) [file pone.0028132.s004.doc]

|  | **Skin** | **Gut** | **Urine** | **Mouth** | **Water** | **Soil** | **Unknown** |
| --- | --- | --- | --- | --- | --- | --- | --- |
| **Ekeley 1 - female** | | | | | | | |
| Door in | 82.14  (0.25) | 0 | 1.23  (0.15) | 5.40  (0.22) | 2.18  (0.11) | 1.21  (0.15) | 7.84  (0.30) |
| Door out | 77.22  (0.53) | 0.03  (0.02) | 1.81  (0.10) | 5.71  (0.32) | 1.67  (0.24) | 0 | 13.56  (0.44) |
| Stall in | 47.89  (0.47) | 0.01  (0.01) | 11.57  (0.24) | 2.43  (0.23) | 10.79  (0.41) | 3.55  (0.14) | 23.76  (0.34) |
| Stall out | 37.82  (0.28) | 0 | 58.50  (0.11) | 0.41  (0.24) | 0 | 0 | 3.27  (0.17) |
| Faucet handle | 95.31  (0.24) | 0.79  (0.07) | 0.18  (0.07) | 0 | 2.05  (0.20) | 0 | 1.67  (0.11) |
| Soap dispenser | 75.42  (0.32) | 0.05  (0.03) | 6.82  (0.19) | 0.03  (0.03) | 3.57  (0.20) | 0.95  (0.15) | 13.16  (0.46) |
| Toilet seat | 31.01  (0.50) | 0 | 18.54  (0.31) | 0 | 0.61  (0.16) | 0 | 49.84  (0.51) |
| Toilet flush handle | 1.58  (0.15) | 0 | 1.22  (0.08) | 0 | 0.88  (0.14) | 0.13  (0.11) | 96.19  (0.17) |
| Toilet floor | 43.90  (0.54) | 0.01  (0.01) | 0.02  (0.01) | 0.01  (0.01) | 0.22  (0.12) | 0.13  (0.09) | 55.71  (0.53) |
| Sink floor | 42.76  (0.41) | 0 | 0.02  (0.01) | 0 | 0.01  (0.01) | 0.02  (0.02) | 57.19  (0.41) |
| **Ekeley 1 – male** | | | | | | | |
| Door in | 75.51  (0.38) | 0 | 0.81  (0.16) | 0.02  (0.02) | 10.61  (0.17) | 0 | 13.05  (0.39) |
| Door out | - | - | - | - | - | - | - |
| Stall in | - | - | - | - | - | - | - |
| Stall out | 94.27  (0.10) | 4.28  (0.12) | 0 | 0.02  (0.02) | 0.21  (0.08) | 0.01  (0.01) | 1.21  (0.14) |
| Faucet handle | 90.33  (0.26) | 0 | 0 | 0 | 0 | 0 | 9.67  (0.26) |
| Soap dispenser | - | - | - | - | - | - | - |
| Toilet seat | 81.08  (0.47) | 0.21  (0.11) | 0.06  (0.05) | 0 | 0.15  (0.10) | 0 | 18.50  (0.42) |
| Toilet flush handle | 55.34  (0.25) | 0.03  (0.03) | 0 | 0 | 16.98  (0.26) | 1.86  (0.21) | 25.79  (0.23) |
| Toilet floor | 68.81  (0.51) | 0.03  (0.03) | 0.01  (0.01) | 0 | 0 | 0.07  (0.04) | 31.08  (0.52) |
| Sink floor | 58.30  (0.71) | 0 | 0 | 0 | 0.15  (0.09) | 0.07  (0.03) | 41.48  (0.67) |
| **Ekeley 2 - female** | | | | | | | |
| Door in | 78.90  (0.28) | 0 | 0.13  (0.07) | 0.07  (0.04) | 4.35  (0.02) | 0.65  (0.19) | 15.90  (0.32) |
| Door out | 80.44  (0.60) | 1.01  (0.11) | 8.69  (0.16) | 2.04  (0.46) | 0.08  (0.04) | 0.01  (0.01) | 7.73  (0.24) |
| Stall in | - | - | - | - | - | - | - |
| Stall out | 94.11  (0.21) | 1.03  (0.07) | 1.78  (0.09) | 0 | 0.01  (0.01) | 0 | 3.07  (0.25) |
| Faucet handle | 76.20  (0.73) | 0.09  (0.05) | 3.74  (0.11) | 0 | 6.87  (0.26) | 0 | 13.10  (0.79) |
| Soap dispenser | 57.98  (0.35) | 28.06  (0.26) | 1.78  (0.13) | 0.40  (0.14) | 3.74  (0.15) | 1.46  (0.14) | 6.58  (0.35) |
| Toilet seat | 10.72  (0.18) | 0 | 85.69  (0.24) | 0.01  (0.01) | 0.02  (0.02) | 0.44  (0.17) | 3.12  (0.26) |
| Toilet flush handle | - | - | - | - | - | - | - |
| Toilet floor | 31.11  (0.32) | 0.03  (0.03) | 1.83  (0.12) | 0 | 0.48  (0.26) | 2.44  (0.22) | 64.11  (0.60) |
| Sink floor | 28.99  (0.52) | 0.02  (0.02) | 0.18  (0.05) | 0.01  (0.01) | 0.54  (0.15) | 0 | 70.26  (0.49) |
| **Ekeley 2 – male** | | | | | | | |
| Door in | - | - | - | - | - | - | - |
| Door out | 86.60  (0.44) | 0.11  (0.05) | 0.04  (0.02) | 0.60  (0.27) | 1.69  (0.19) | 0.03  (0.02) | 10.93  (0.15) |
| Stall in | 80.05  (0.45) | 0 | 0.78  (0.28) | 0.20  (0.11) | 8.64  (0.26) | 0.01  (0.01) | 10.32  (0.31) |
| Stall out | 88.80  (0.38) | 0.09  (0.05) | 2.01  (0.18) | 5.03  (0.33) | 1.25  (0.18) | 0.20  (0.09) | 2.62  (0.25) |
| Faucet handle | 88.33  (0.27) | 0.06  (0.03) | 7.34  (0.19) | 0.31  (0.12) | 1.48  (0.19) | 0 | 2.48  (0.22) |
| Soap dispenser | 81.34  (0.40) | 0.05  (0.04) | 0.24  (0.09) | 2.25  (0.34) | 6.56  (0.14) | 0 | 9.56  (0.35) |
| Toilet seat | 8.04  (0.25) | 46.91  (0.34) | 1.57  (0.10) | 0.03  (0.03) | 0.37  (0.19) | 0 | 43.08  (0.39) |
| Toilet flush handle | 72.27  (0.37) | 0.01  (0.01) | 2.18  (0.14) | 0 | 2.72  (0.27) | 0.08  (0.06) | 22.74  (0.44) |
| Toilet floor | 42.78  (0.38) | 0 | 0 | 0.03  (0.02) | 1.09  (0.14) | 0.95  (0.16) | 55.15  (0.36) |
| Sink floor | 51.99  (0.58) | 0 | 0.02  (0.01) | 0 | 1.05  (0.08) | 0.01  (0.01) | 46.93  (0.55) |
| **Ekeley 3 – female** | | | | | | | |
| Door in | 74.98  (0.43) | 0 | 1.47  (0.15) | 5.95  (0.30) | 3.37  (0.24) | 1.98  (0.10) | 12.25  (0.29) |
| Door out | 41.97  (0.62) | 0.01  (0.01) | 1.78  (0.08) | 21.68  (0.33) | 0 | 0 | 34.56  (0.51) |
| Stall in | - | - | - | - | - | - | - |
| Stall out | 90.48  (0.36) | 0.01  (0.01) | 0.01  (0.01) | 0.07  (0.05) | 4.96  (0.17) | 0.03  (0.02) | 4.44  (0.30) |
| Faucet handle | 46.70  (0.63) | 0.01  (0.01) | 2.71  (0.16) | 0.76  (0.27) | 0 | 0 | 49.82  (0.35) |
| Soap dispenser | 33.62  (0.35) | 0.13  (0.08) | 57.93  (0.28) | 0.03  (0.02) | 0.04  (0.03) | 0.02  (0.01) | 8.23  (0.26) |
| Toilet seat | 31.27  (0.41) | 15.71  (0.31) | 17.40  (0.31) | 0 | 0 | 0.17  (0.09) | 35.45  (0.73) |
| Toilet flush handle | 31.06  (0.36) | 21.31  (0.25) | 27.86  (0.30) | 0 | 13.76  (0.18) | 0 | 6.01  (0.25) |
| Toilet floor | 45.17  (0.81) | 0.09  (0.08) | 0.27  (0.09) | 0 | 4.49  (0.51) | 0.33  (0.14) | 49.65  (0.79) |
| Sink floor | 45.87  (0.55) | 0 | 0.12  (0.07) | 0 | 1.87  (0.24) | 2.19  (0.20) | 49.95  (0.63) |
| **Ekeley 3 – male** | | | | | | | |
| Door in | 80.91  (0.27) | 0.01  (0.01) | 0.08  (0.05) | 0.25  (0.16) | 7.00  (0.17) | 0.05  (0.05) | 11.70  (0.23) |
| Door out | 81.55  (0.31) | 2.22  (0.15) | 0.46  (0.10) | 11.10  (0.20) | 1.55  (0.09) | 0.04  (0.03) | 3.08  (0.28) |
| Stall in | - | - | - | - | - | - | - |
| Stall out | 96.78  (0.24) | 0 | 0.01  (0.01) | 0.03  (0.02) | 0.03  (0.03) | 0 | 3.15  (0.24) |
| Faucet handle | 90.63  (0.28) | 0.13  (0.04) | 0.01  (0.01) | 7.76  (0.35) | 0.02  (0.01) | 0.01  (0.01) | 1.44  (0.14) |
| Soap dispenser | - | - | - | - | - | - | - |
| Toilet seat | 40.00  (0.39) | 33.87  (0.29) | 0.11  (0.07) | 0.01  (0.01) | 0.10  (0.07) | 0.03  (0.02) | 25.88  (0.25) |
| Toilet flush handle | 14.17  (0.19) | 78.63  (0.13) | 0.24  (0.08) | 0.01  (0.01) | 0.03  (0.02) | 0.01  (0.01) | 6.91  (0.22) |
| Toilet floor | 54.74  (0.48) | 0.02  (0.01) | 0.01  (0.01) | 0 | 0.01  (0.01) | 0.07  (0.06) | 45.15  (0.47) |
| Sink floor | 62.55  (0.62) | 0 | 0 | 0 | 0.26  (0.20) | 0 | 37.19  (0.63) |
| **Porter 1 – female** | | | | | | | |
| Door in | 81.94  (0.31) | 0 | 2.16  (0.11) | 0.19  (0.16) | 4.92  (0.17) | 1.20  (0.13) | 9.59  (0.30) |
| Door out | 78.49  (0.35) | 0.02  (0.02) | 5.62  (0.16) | 0.01  (0.01) | 3.16  (0.31) | 0.02  (0.02) | 12.68  (0.44) |
| Stall in | - | - | - | - | - | - | - |
| Stall out | - | - | - | - | - | - | - |
| Faucet handle | 76.32  (0.35) | 1.28  (0.11) | 3.30  (0.19) | 9.69  (0.23) | 0 | 0 | 9.41  (0.37) |
| Soap dispenser | 77.91  (0.35) | 0 | 1.53  (0.17) | 0.04  (0.03) | 4.53  (0.30) | 0.01  (0.01) | 15.98  (0.51) |
| Toilet seat | 11.22  (0.28) | 63.07  (0.30) | 9.35  (0.12) | 0 | 0.05  (0.03) | 0 | 16.31  (0.42) |
| Toilet flush handle | 52.98  (0.38) | 0 | 31.78  (0.22) | 0 | 0.06  (0.04) | 0.01  (0.01) | 15.17  (0.34) |
| Toilet floor | 36.67  (0.26) | 2.14  (0.08) | 0.30  (0.12) | 0 | 0.27  (0.11) | 0 | 60.62  (0.33) |
| Sink floor | 27.00  (0.65) | 3.38  (0.17) | 1.48  (0.10) | 0 | 0.78  (0.14) | 0.09  (0.06) | 67.27  (0.68) |
| **Porter 1 – male** | | | | | | | |
| Door in | - | - | - | - | - | - | - |
| Door out | 66.23  (0.39) | 0 | 2.44  (0.21) | 0.66  (0.18) | 14.67  (0.18) | 0.07  (0.03) | 15.93  (0.43) |
| Stall in | 64.45  (0.36) | 11.42  (0.29) | 0.01  (0.01) | 0 | 6.34  (0.33) | 0.22  (0.11) | 17.56  (0.42) |
| Stall out | 91.49  (0.36) | 0.04  (0.03) | 0 | 0 | 2.11  (0.11) | 0 | 6.35  (0.35) |
| Faucet handle | 91.91  (0.23) | 0 | 0.03  (0.02) | 6.65  (0.20) | 0.01  (0.01) | 0 | 1.40  (0.15) |
| Soap dispenser | 87.56  (0.38) | 0 | 0.06  (0.05) | 6.87  (0.24) | 2.16  (0.10) | 0.22  (0.10) | 3.13  (0.22) |
| Toilet seat | 47.30  (0.43) | 25.23  (0.31) | 0 | 0 | 0.47  (0.20) | 0.03  (0.03) | 26.97  (0.41) |
| Toilet flush handle | 52.45  (0.48) | 23.73  (0.28) | 0.64  (0.24) | 0 | 2.66  (0.14) | 0.05  (0.05) | 20.47  (0.53) |
| Toilet floor | 26.17  (0.52) | 10.47  (0.15) | 0.51  (0.09) | 0.03  (0.03) | 0.94  (0.11) | 0.12  (0.06) | 61.76  (0.61) |
| Sink floor | 23.94  (0.42) | 0.01  (0.01) | 0.46  (0.08) | 0 | 0.07  (0.04) | 0.12  (0.08) | 75.40  (0.38) |
| **Porter 2 – female** | | | | | | | |
| Door in | - | - | - | - | - | - | - |
| Door out | 62.54  (0.28) | 0 | 1.58  (0.13) | 0.02  (0.01) | 10.02  (0.35) | 4.24  (0.15) | 21.60  (0.48) |
| Stall in | - | - | - | - | - | - | - |
| Stall out | - | - | - | - | - | - | - |
| Faucet handle | - | - | - | - | - | - | - |
| Soap dispenser | 71.66  (0.60) | 0 | 6.47  (0.21) | 3.58  (0.31) | 2.06  (0.17) | 0.02  (0.01) | 16.21  (0.34) |
| Toilet seat | 3.90  (0.21) | 87.84  (0.19) | 3.33  (0.10) | 2.31  (0.18) | 2.55  (0.10) | 0.05  (0.03) | 0.02  (0.01) |
| Toilet flush handle | - | - | - | - | - | - | - |
| Toilet floor | 35.31  (0.49) | 0.05  (0.03) | 0.57  (0.15) | 0 | 0.03  (0.02) | 0.03  (0.02) | 64.01  (0.42) |
| Sink floor | 24.36  (0.49) | 0 | 0.40  (0.15) | 0 | 0.17  (0.09) | 0.08  (0.07) | 74.99  (0.43) |
| **Porter 2 – male** | | | | | | | |
| Door in | - | - | - | - | - | - | - |
| Door out | 81.82  (0.28) | 0 | 0 | 0.02  (0.02) | 9.88  (0.17) | 0.30  (0.17) | 7.98  (0.29) |
| Stall in | 36.42  (0.50) | 0 | 0 | 2.11  (0.21) | 0.98  (0.31) | 0.02  (0.01) | 60.47  (0.56) |
| Stall out | 78.09  (0.31) | 0 | 0.02  (0.02) | 0 | 7.29  (0.20) | 0.05  (0.03) | 14.55  (0.32) |
| Faucet handle | 74.73  (0.33) | 0 | 2.62  (0.17) | 0.04  (0.03) | 2.32  (0.32) | 0.04  (0.02) | 20.25  (0.49) |
| Soap dispenser | 92.20  (0.16) | 0.01  (0.01) | 0.09  (0.05) | 0.07  (0.04) | 3.10  (0.19) | 0.03  (0.02) | 4.50  (0.32) |
| Toilet seat | - | - | - | - | - | - | - |
| Toilet flush handle | 21.52  (0.26) | 45.95  (0.30) | 0 | 0.01  (0.01) | 0.77  (0.28) | 0.45  (0.09) | 31.30  (0.46) |
| Toilet floor | 36.56  (0.37) | 4.62  (0.17) | 0.18  (0.09) | 0 | 2.67  (0.26) | 2.42  (0.23) | 53.55  (0.47) |
| Sink floor | 33.22  (0.49) | 0.08  (0.04) | 0.03  (0.02) | 0 | 0.03  (0.02) | 0.10  (0.04) | 66.54  (0.48) |
| **Porter 3 - female** | | | | | | | |
| Door in | 87.20  (0.26) | 0.04  (0.02) | 6.79  (0.16) | 0.25  (0.16) | 0.01  (0.01) | 0.02  (0.02) | 5.69  (0.24) |
| Door out | 70.09  (0.42) | 0 | 18.71  (0.25) | 0.31  (0.13) | 0.01  (0.01) | 0.01  (0.01) | 10.87  (0.40) |
| Stall in | 68.47  (0.40) | 0.06  (0.02) | 8.95  (0.16) | 1.35  (0.35) | 4.50  (0.23) | 0.13  (0.07) | 16.54  (0.41) |
| Stall out | 86.49  (0.43) | 0 | 3.17  (0.12) | 1.62  (0.34) | 3.67  (0.13) | 0.07  (0.04) | 4.98  (0.26) |
| Faucet handle | 1.34  (0.19) | 0 | 0.04  (0.02) | 0 | 0 | 0 | 98.62  (0.18) |
| Soap dispenser | 58.83  (0.43) | 0 | 11.46  (0.19) | 16.60  (0.27) | 2.52  (0.09) | 0.43  (0.10) | 10.16  (0.30) |
| Toilet seat | 51.87  (0.39) | 7.10  (0.19) | 31.63  (0.32) | 0 | 0.01  (0.01) | 0 | 9.39  (0.47) |
| Toilet flush handle | 48.54  (0.45) | 0 | 0.98  (0.19) | 0.01  (0.01) | 19.64  (0.35) | 0 | 30.83  (0.42) |
| Toilet floor | 53.35  (0.68) | 0.06  (0.04) | 3.18  (0.12) | 0.15  (0.08) | 0.02  (0.01) | 0.43  (0.16) | 42.81  (0.72) |
| Sink floor | 44.53  (0.59) | 1.17  (0.14) | 1.17  (0.08) | 0 | 0.03  (0.02) | 0.62  (0.22) | 52.48  (0.73) |
| **Porter 3 – male** | | | | | | | |
| Door in | 78.49  (0.26) | 0 | 0.01  (0.01) | 0.28  (0.14) | 0 | 0.01  (0.01) | 21.21  (0.21) |
| Door out | 85.41  (0.42) | 0.04  (0.03) | 0.01  (0.01) | 3.16  (0.20) | 0.54  (0.04) | 0 | 10.84  (0.29) |
| Stall in | 58.06  (0.38) | 23.09  (0.18) | 0 | 4.33  (0.24) | 1.81  (0.10) | 0.01  (0.01) | 12.70  (0.36) |
| Stall out | 92.21  (0.34) | 0 | 0.03  (0.02) | 0 | 0 | 0 | 7.76  (0.34) |
| Faucet handle | 0.46  (0.20) | 0 | 0 | 0 | 0.01  (0.01) | 0 | 99.53  (0.20) |
| Soap dispenser | 30.31  (0.28) | 0 | 0.01  (0.01) | 2.16  (0.23) | 7.16  (0.51) | 1.53  (0.19) | 58.83  (0.54) |
| Toilet seat | 21.56  (0.34) | 53.66  (0.27) | 0.06  (0.06) | 0.01  (0.01) | 0.64  (0.28) | 0.07  (0.04) | 24.00  (0.41) |
| Toilet flush handle | 38.31  (0.25) | 46.18  (0.20) | 0 | 0 | 3.31  (0.16) | 0.02  (0.01) | 12.18  (0.29) |
| Toilet floor | 15.12  (0.52) | 56.95  (0.44) | 0.02  (0.01) | 0.03  (0.03) | 1.60  (0.17) | 0 | 26.28  (0.84) |
| Sink floor | 47.69  (0.46) | 0 | 1.40  (0.13) | 0 | 1.21  (0.14) | 2.52  (0.16) | 47.18  (0.48) |
